# Structural Analysis of Human Fascin-1: Essential Protein for Actin Filaments Bundling

**DOI:** 10.3390/life12060843

**Published:** 2022-06-06

**Authors:** Jeong Min Chung, Osamu Sato, Reiko Ikebe, Sangmin Lee, Mitsuo Ikebe, Hyun Suk Jung

**Affiliations:** 1 Department of Biotechnology, The Catholic University of Korea, Bucheon-si 14662, Gyeonggi, Korea; jmchung@catholic.ac.kr; 2 Department of Cellular and Molecular Biology, The University of Texas Health Science Center at Tyler, 11937 US Highway 271, Tyler, TX 75708, USA; osamu.sato@uthct.edu (O.S.); reiko.ikebe@uthct.edu (R.I.); 3 Department of Biochemistry, College of Natural Sciences, Kangwon National University, Chuncheon 24341, Gangwon, Korea

**Keywords:** fascin, actin-binding, actin-bundling, actin-binding site, electron microscopy

## Abstract

Fascin, a major actin cross-linking protein, is expressed in most vertebrate epithelial tissues. It organizes actin filaments into well-ordered bundles that are responsible for the extension of dynamic membrane protrusions, including microspikes, filopodia, and invadopodia from cell surfaces, which are involved in cell migration and invasion as critical components of cancer metastasis. However, it is not well-understood how fascin-1 induces actin binding/bundling and where fascin-1 localizes along the actin filaments, thus facilitating actin bundle formation. In the present study, we attempted to clarify these problems by using biochemical and electron microscopic analyses using various fascin-1 constructs. Three dimensional structures of actin/fascin-1 complex were obtained by electron microscopy (EM) with iterative helical real-space reconstruction (IHRSR) and tomography. We revealed that the N-terminal region containing the Actin-Binding Site 2 (ABS2) of fascin-1 is responsible for actin bundling and the C-terminal region is important for the dimerization of fascin-1. We also found that the dimerization of fascin-1 through intermolecular interactions of the C-terminal region is essential for actin bundling. Since fascin is an important factor in cancer development, it is expected that the findings of present study will provide useful information for development of therapeutic strategies for cancer.

## 1. Introduction

Actin filaments exist in three major structures in cells: single filaments, meshworks, and a cross-linked network of filaments called bundles [1]. The formation of actin meshworks or bundles requires the presence of various types of actin-binding proteins [1]. Among them, the actin crosslinking proteins that play an important role in regulating such actin structures are divided into two large groups: one that possess at least two actin-binding sites and the other acting as a dimer.

In general, the actin crosslinking proteins forming meshworks of actin filaments have high molecular weight with elongated structures such as filamin, while the crosslinking proteins that facilitate actin bundle formation are small with globular shapes such as fascin and ABP34 [1,2,3]. Fascin is a 55 kDa actin-bundling protein that was originally identified in unfertilized sea urchin eggs [3] and is the major actin crosslinking protein contributing to the protrusive cellular structures, including filopodia, dendrites, and invadopodia, which are the key structures of various cellular functions such as cell motility, cellular protection, and invasion to the extracellular matrix [4,5]. Fascin has two actin-binding sites (ABS) at each end of the protein (C-terminal and N-terminal domains), and it binds to F-actin in a 1:2 (in brain tissue) or 1:4 (in vitro) ratio [6,7,8,9]. The actin-bundling activity of fascin is regulated by protein kinase Cα (PKCα), which inhibits fascin’s ability to bind actin at both sites [6,10]. Although the binding of fascin to actin has been documented by several studies, there is no structure of the F-actin–fascin complex that can determine the precise structures of the actin-binding sites at the atomic level. This is critical missing information needed to understand the fascin-dependent regulation of bundling formation, which is ultimately required to understand the cellular distribution of fascin and actin filament assemblies in cells [4,6].

In this paper, we investigated the molecular and structural basis of fascin-dependent actin-bundling formation by co-sedimentation assays and electron microscopic (EM) analysis using various fascin constructs, namely full length (FLF), N-terminal half (NHF), C-terminal half (CHF), and Leucine zipper peptide-fused full-length fascin (LZFLF). Our results revealed that the dimerization of fascin is crucial for the fascin-mediated cross-linking of actin filaments. Moreover, our structural analysis provided the molecular basis of the actin-binding nature of the actin-binding site 2 (ABS2) of fascin. 

## 2. Materials and Methods

### 2.1. Making Constructs and the Purification of the Recombinant WT Fascin and Its Mutants

Recombinant full-length human fascin 1 (GenBank accession number: NM003088.4) was obtained from human spleen cDNA library (including 1–493rd fascin amino acids). To create the FLF construct, the Fascin cDNA flanking the BamHI/KpnI restriction enzyme sites was subcloned into a modified pFastBacHT baculovirus transfer vector (Thermo Fisher) containing a FLAG sequence. To create NHF (amino acid residues 1–259) and CTD12 constructs (amino acid residues 1–481), the stop codons were inserted at 3′ side of 777th cytosine and 1443th guanine, respectively, by PCR site-directed mutagenesis [11] using Pfu Ultra II (Agilent Technologies). For the CHF construct (amino acid residues 260–493), a BamHI site was created in place of 772–777th Fascin cDNA, and 5′-side half of the Fascin cDNA sequence was removed by BamH1 restriction enzyme digestion. For FLF-LZ construct, an EcoRI site was created in place of the stop codon in the FLF construct, and a BamHI/EcoRI fragment containing the FLF sequence was introduced into a modified pFastHT/FLAG vector containing the leucine zipper sequence [12]. 

The sequence of FLF-LZ after the 493rd amino acid was EFGPTRGGGSGGGSGGGSSMKQLEDKVEEL LSKNYHLENEVARLKKLVGERTS. Fascin proteins were expressed in Sf9 cells by baculovirus expression system as described in the manufacturer’s protocol (Invitrogen) and purified using Ni-NTA column chromatography or anti-FLAG antibodies affinity column chromatography as described previously [13]. In short, 5 × 10^8^ Sf9 cells were infected with fascin-expressing virus and harvested after 3 days. The packed cells were homogenized with 0.1 M NaCl, 30 mM Tris-HCl (pH 8), 10 mM EGTA, 1 mM dithiothreitol (DTT), 0.1 mM PMSF, and 10 µg/mL leupeptin and centrifuged at 150,000× *g* for 20 min. The supernatant was mixed with 0.3 mL Ni-NTA or 0.4 mL anti-FLAG antibodies agarose resins and rotated for 1 h at 4 °C. After two washes with a buffer containing 0.3 M NaCl, 10 mM imidazole-HCl (pH 7.5), 0.2 mM EGTA, 1 µg/mL leupeptin and 1 mM DTT or 0.15 M NaCl, 20 mM MOPS-KOH (pH 7.5), 1 mM EGTA, 1 µg/mL leupeptin, and 0.1 mM DTT, the protein was eluted with the elution buffer containing 200 mM imidazole-HCl (pH7.5) or 0.1 mM FLAG peptide, respectively. The proteins were flash-frozen by liquid N_2_ in the presence of 15% sucrose and stored at −80 °C until use.

### 2.2. F-Actin Bundling and Binding Assay

Actin-bundling activity was measured by a low-speed co-sedimentation assay and negative staining electron microscopy as described previously [2]. High-speed co-sedimentation assay (actin-binding assay) was performed via the method described by Fechheimer and Taylor [14] and Fechheimer [15]. G-actin (3 µM) was mixed with variants (15 μM) in F-actin buffer to a final volume of 120 μL, and 20 μL of this mixture was taken out as the total sample. The remaining 100 μL of mixture was held at 37 °C for 2 h, after which the mixture was centrifuged in an airfuge (Beckman, USA) at 115,000× *g* for 30 min. The supernatant solution (S) was collected, and the pellet (P) was resuspended in SDS–PAGE sample buffer. The supernatant solution was diluted to the same volume as was the pellet with the sample buffer. Equal volumes of the both supernatant and pellet fractions were analyzed on Coomassie-stained SDS–PAGE gels.

### 2.3. Data Collection Using Transmission Electron Microscope

A sample of 5 μL was applied to glow discharged grid firmly held by self-closing tweezers (Dumont, Swiss), and 3–4 drops of 1% aqueous uranyl acetate was applied to the grid. After 1 min reaction time, the excess stain solution was then removed by touching the edge of the grid with filter paper (Whatman, UK). The grid was then dried using hair dryer for 10 s. The prepared grids were examined in a Technai 10 TEM (FEI, USA) operated at 100 kV, and the images were recorded on an Ultrascan 1000 CCD camera (Gatan, USA) at a magnification of 0.32 nm/pixel for both single-particle analysis and electron tomography. Instrumentation was used in Kangwon Center for Systems Imaging. For negative staining electron tomography, the sample tilts around a single axis from ±60° with 2° intervals to obtain a tilt series.

### 2.4. Image Processing

For the single particle analysis of fascin dimer, the images of individual particles were selected from the raw micrographs, windowed out, and imported into the SPIDER program. A total of 1200 full-length fascin particles were used for processing, and the class averages were produced with the reference-free method as described [16]. The USCF Chimera program was used for visualization and comparative analyses of the atomic models and averages. For the 3D reconstruction of F-actin/fascin model, single particle with the iterative helical real-space reconstruction (IHRSR) approach [17] was carried out as described in [18]. Projection matching was performed against pure F-actin atomic model, which was filtered to 2 nm resolution. The uniformly decorated 3800 segments were used to generate a 3D reconstruction of fascin-decorated F-actin. For electron tomography, the collected 60 projections of tilt series were aligned based on the fiducial markers (5 nm gold nanoparticles) and reconstructed through Radon transform with cone beam geometry using least squares based filtered back-projection in the IMOD etomo module (IMOD software package) [19,20]. The resulting 3D density map was displayed and examined using UCSF Chimera software [21].

## 3. Results

### 3.1. Two-Major Actin-Binding Sites of Fascin

**Figure 1:**
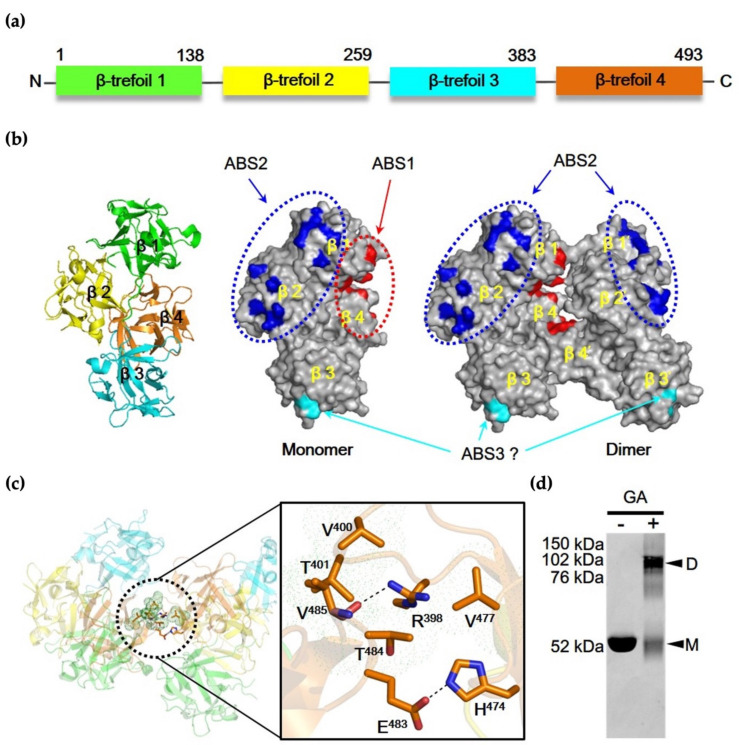
Two major actin-binding sites of Fascin. (**a**) Schematic diagram of human fascin, consisting of four β-trefoil domains. Each domain is highlighted in a different color. (**b**) Proposed model for F-actin-binding sites in fascin crystal structure (adapted from PDB code: 3LLP [24]), built from atomic model of fascin crystal structure. Surface presentation of the fascin crystal structure showing the two major and one minor actin-binding sites. Two forms of surface view show the monomer and dimer structures, respectively. Binding site 1 (red) consists of β-trefoil domain 1 and 4, is located between N- and C-termini, and is situated within interface of dimer. Binding site 2 (blue) consisting of β-trefoil domain 1 and 2 and is located on outer surface of dimer. Binding site 3 (cyan) in the β-trefoil domain 3 is a newly suggested binding site. The dashed circles (red and blue) indicate major F-actin-binding site 1 and 2. (**c**) Ribbon representation of the dimeric structure of fascin, which is composed of NC::CN form. The boxed region shows the interactions between dimer interfaces. Dashed lines and dotted circles indicate polar interaction and hydrophobic interaction. (**d**) Chemical cross-linking experiments using 0.1% GA. D and M represent dimer and monomer, respectively.

The fascin, a 55 kDa protein, consists of 4 β-trefoil folds (β1-domain–β4-domain), which pack to form a distorted tetrahedron organized in two lobes with a skew angle of approximately 56° (Figure 1a) [22]. Although the structure of fascin–F-actin complex at high enough resolution to allow mapping the precise actin-binding sites is not available, it is thought that the fascin protein has at least two actin-binding sites (ABS1 and ABS2) since fascin monomer crosslinks F-actin filaments to produce actin bundling [9,23]. Based on previous structural and biochemical studies, we proposed schematic models of the monomer and dimer of fascin [24,25] (Figure 1b).

**Figure 2:**
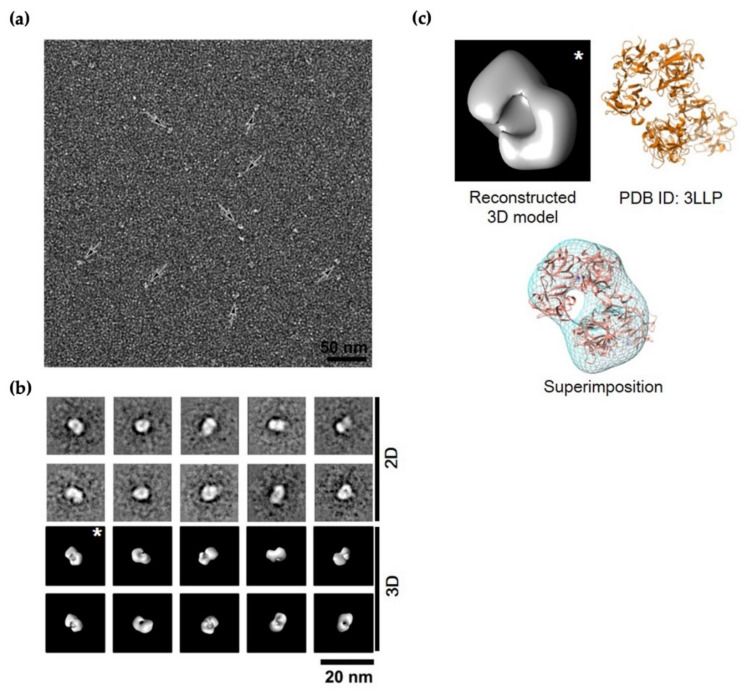
Appearances of fascin visualized by electron microscopy followed by single-particle analysis. (**a**) Negatively stained fields of full-length fascin. Black arrows indicate individual fascin molecules. (**b**) Representative 2D class average images and reconstructed 3D structure model of human fascin. (**c**) Fitting of atomic model (adapted from PDB ID: 3LLP [24]) to the 3D electron density map (left panel, asterisk-marked average in (**b**)). Scale bars indicate 50 nm and 20 nm in (**a**,**b**), respectively.

The monomeric form of the model showed that fascin contains three actin-binding sites. The first actin-binding site (ABS1) is formed by amino acid residues from the N-termini (β-trefoil domain 1, β1-domain) and C-termini (β-trefoil domain 4, β4-domain), while the second actin-binding site (ABS2) is located at opposite side of ABS1 (Figure 1b). The ABS2 covers residues from β1-domain and β2-domain. The location and shape of the two actin-binding sites, sustaining the open form of fascin protein, may facilitate the actin-bundling activity of the monomeric form. Recently, the third binding site (ABS3), which is formed by amino acid residues from β-trefoil domain 3 (β3-domain), was predicted as novel actin-binding site of fascin protein, albeit with an unclear role in the actin-binding activity [23]. Unlike the monomeric model, the dimeric model of fascin illustrated that the amino acids of ABS1 are buried between the dimeric interfaces, implying that the ABS1 may not play a crucial role in actin-binding function in the fascin dimer (Figure 1b). In contrast to ABS1, ABS2 of each fascin subunit of the fascin dimer was exposed on the outer surfaces of the dimer. Taken together, both ABS1 and ABS2 are important for the actin binding in monomeric form of the fascin protein, while the fascin dimer only uses ABS2 for actin-binding activity. Within the dimeric interface, two types of inter-molecular interactions were proposed: the polar interaction and the hydrophobic interaction (Figure 1c). The polar interaction is formed by Val485 and Glu483 in the β4-domain of one fascin monomer and Arg398 and His474 in the β4-domain of the other monomer, respectively. On the other hand, the hydrophobic core is formed by the interaction between Thr401 and Thr484 in β4-domain of one monomer and Val400 and Val477 in β4-domain of the other, resulting in the formation of N-term-C-term to C-term-N-term (NC::CN) dimeric complex. To confirm whether the fascin dimer exists in vitro, a chemical cross-linking experiment was conducted using 0.1% glutaraldehyde (GA) (Figure 1d). The native gel analysis of the cross-linked product showed two bands that are consistent with the molecular masses of monomer and dimer, respectively. The result suggested that both fascin monomer and dimer exist in vitro. This is supported by the negative staining EM single-particle analysis. As shown in Figure 2, the population ratio of monomer and dimer in negatively stained full-length fascin particles on EM micrographs was almost equal to 1 (at 1:1 ratio). Moreover, the dimeric crystal structure of fascin (PDB ID: 3LLP [24]) was well superimposed to the 3D electron density map (Figure 2c). All these data suggested that the fascin can exist as both monomeric and dimeric forms. 

### 3.2. Actin-Bundling Activity of Fascin and Its Truncated Mutants

**Figure 3:**
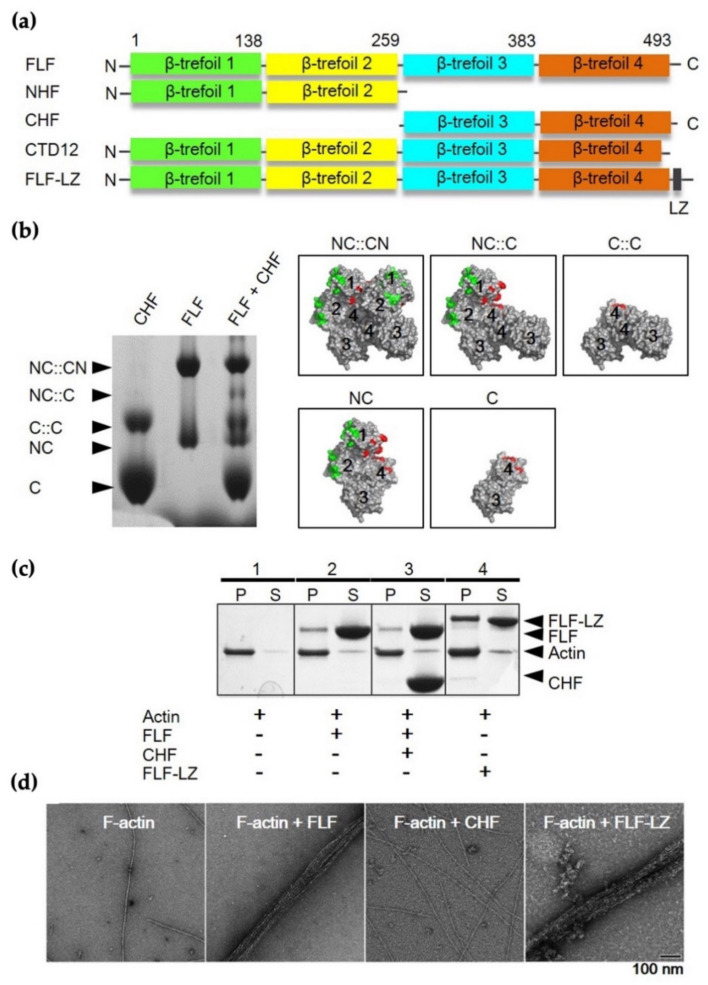
Construction of various deletion constructs and molecular characterization of each domain. (**a**) Production of various deletion mutants and leucine zipper-containing constructs. FLF, full-length of human fascin; NHF, C-terminal domain deletion (beta-trefoil 3 and 4); CHF, N-terminal domain deletion (beta-trefoil 1 and 2); CTD, 12 amino acids deletion at the C-terminal of the beta-trefoil domain4; FLF-LZ, leucine zip sequence addition at the C-terminus. (**b**) Dimerization test of human fascin using 8% native PAGE. Equal concentrations (2.5 μg) of FLF and CHF were mixed and incubated for 1 h at 4 °C. Lane 1, CHF; lane 2, FLF; lane 3, FLF + CHF. The right panel shows the model of the expected oligomeric formation. NC::CN, dimeric human fascin; NC::C, binding of monomeric FLF and CHF; C::C, dimerization of CHF; NC, monomeric FLF; C, monomeric CHF. (**c**) Actin-binding assay. For actin/fascin co-sedimentation analysis, three micromoles of G-actin and fascin variant were mixed and incubated at 4 °C for 1 h, and then, the actin/fascin mixtures were separated into supernatant (S) and pellet (P) and loaded onto 12.5% SDS–PAGE. Lane 1, actin alone; Lane 2, actin + FLF; Lane 3, actin + FLF + CHF; Lane 4, actin + CHF; Lane 5, Actin + FLF-LZ. (**d**) Actin-bundling activity using negative staining EM analysis for each mutant of fascin. Scale bar indicates 100 nm.

To analyze the actin-bundling activity of fascin, several recombinant constructs were produced, including full-length (FLF), N-terminal half (NHF), C-terminal half (CHF), C-terminal end deletion (CTD12), and FLF with leucine zipper sequence at the C-terminus (FLF-LZ) (Figure 3a). The N-terminal half construct includes β1 and β2-trefoil domain (residues 1–259), while the C-terminal half construct involves β3 and β4-trefoil domain (residues 260–493). The C-terminal deletion construct contains 481 amino acids without the C-terminal 12 amino acids implicated in dimerization. To investigate the dimerization properties of each construct, native PAGE analysis was performed (Figure 3b). In this experiment, equal amounts (2.5 μg) of FLF and CHF were mixed and then incubated for 1h at 4 °C. Four possible dimeric forms were produced, as both constructs contain the dimerization domain. The expected oligomer structures are shown in the right panel of Figure 3b. The CHF mutant resulted in a band on the SDS–PAGE gel corresponding to the size of C-half-C-half (C::C) complex (Figure 3b). This result suggests that the C-domain is involved in the dimerization of fascin.

The actin-binding and -bundling activities of the constructs were assessed by using high-speed co-sedimentation with G-actin and negative staining EM analysis with F-actin, respectively (Figure 3c,d).

As expected, the FLF showed actin-binding activity (Figure 3c) and actin-bundling activity yielding well-ordered actin bundles (Figure 3d). On the other hand, CHF did not show either actin-binding or -bundling activities, implying that the β3 and β4-trefoild domain is not critical for both actin-binding and -bundling activities. Furthermore, it should be noted that the artificially synthesized dimeric form of fascin (FLF-LZ) showed actin-binding and bundling activities, indicating that dimerization may be important for the actin-bundling activity of fascin, which is contrary to previous studies.

### 3.3. Effect of Dimerization on Actin-Bundling Activity of Fascin

**Figure 4:**
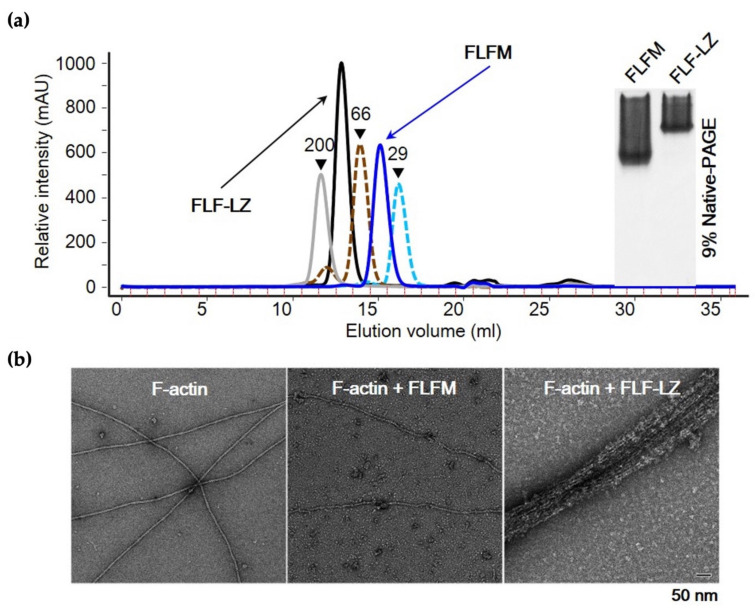
Actin-bundling assay for monomer (FLFM) and dimer (FLFD) of full-length fascin. (**a**) Analytical gel-filtration profile (left) and native PAGE (right) of FLFM (blue line) and FLF-LZ (black line). The positions of the molecular-weight markers are indicated by dashed cyan line (carbonic anhydrase, 29 kDa), dashed brown line (bovine serum albumin, 66 kDa), and gray line (amylase from sweet potato, 200 kDa). (**b**) Negatively stained EM micrographs showing actin-bundling activity of FLFM and FLF-LZ. The scale bar represents 50 nm.

To study the importance of dimerization in the actin-bundling activity of fascin, recombinant full-length fascin (FLF) was subjected to gel filtration to purify the monomeric fraction from the monomer/dimer mixed population. As shown in the size exclusion gel filtration and native PAGE data in Figure 4a, the FLF monomer (FLFM) was well-separated. In addition, the molecular mass of synthesized dimeric form of fascin (FLF-LZ) determined by gel filtration was 110,000 Da, indicating that the construct is properly generated as the dimer form. Surprisingly, the monomer form of FLF (FLFM) did not activate actin-bundling activity, while the dimer form of FLF (FLF-LZ) induced the formation of actin bundles (Figure 4b).

**Figure 5:**
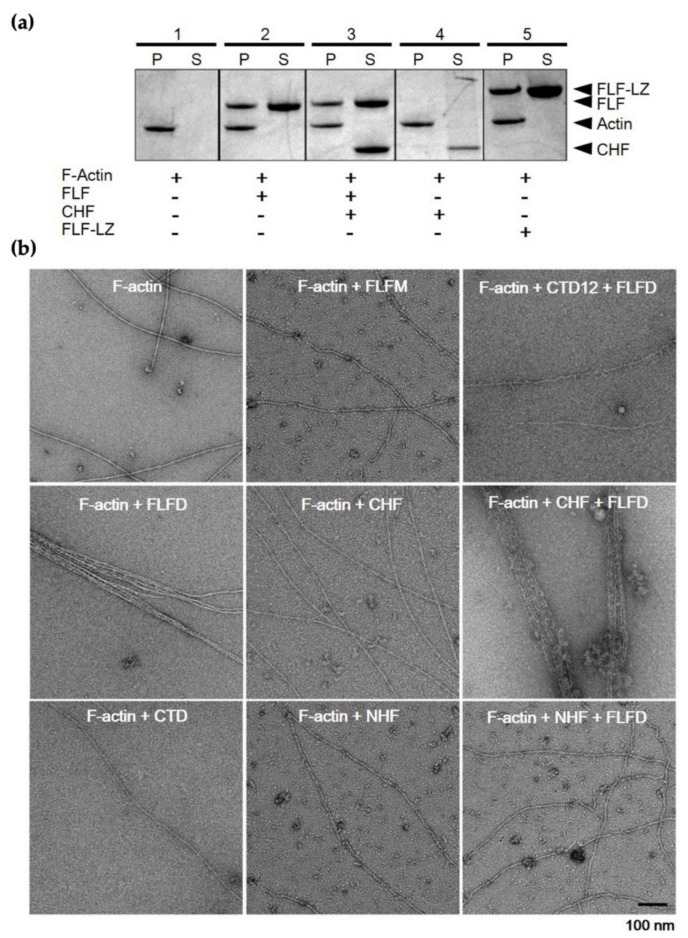
Involvement of dimerization in actin-bundling activity of fascin. (**a**) F-actin co-sedimentation assays illustrating the actin-bundling activities of various fascin constructs. (**b**) Negative staining EM analysis for actin-bundling activity of the mutants and mixture of constructs. FLFM, full-length of human fascin monomer; FLFD, full-length of human fascin dimer; NHF, C-terminal domain deletion (beta-trefoil 3 and 4); CHF, N-terminal domain deletion (beta-trefoil 1 and 2); CTD, 12 amino acids deletion at the C-terminal of the beta-trefoil domain4; FLF-LZ, leucine zip sequence addition at the C-terminus. The scale bar shows 100 nm.

Further investigation of effect of dimerization on the actin-bundling activity was performed with various truncated mutants (Figure 5). In accordance with previous data, the FLFM and CHF showed impaired actin-bundling activities, while FLFD induced actin bundles. In addition, the of C-terminal deletion mutant (CTD12) lacking dimerization showed impaired F-actin-bundling activity. Interestingly, when FLFD was incubated with F-actin/CHF mixture, the actin-bundling activity was inhibited. In contrast, the actin bundling was augmented when FLFD was added into F-actin/NHF mixture (Figure 5b). These results imply that N-domain but not C-domain is important for actin binding. In other words, the actin-binding site 2 (ABS2) consisting of β1 and β2-trefoil domain is crucial for actin binding, whereas ABS1 residing in C-domain is not involved in actin binding. Taken together, dimerization of fascin is the key feature for the actin-bundling activity of this protein, and the ABS1 is crucial for the dimerization of the protein. Furthermore, the C-domain may not be involved in either actin binding or bundling but rather dimerization of the protein.

### 3.4. F-Actin-Binding and -Bundling Models of Fascin

**Figure 6:**
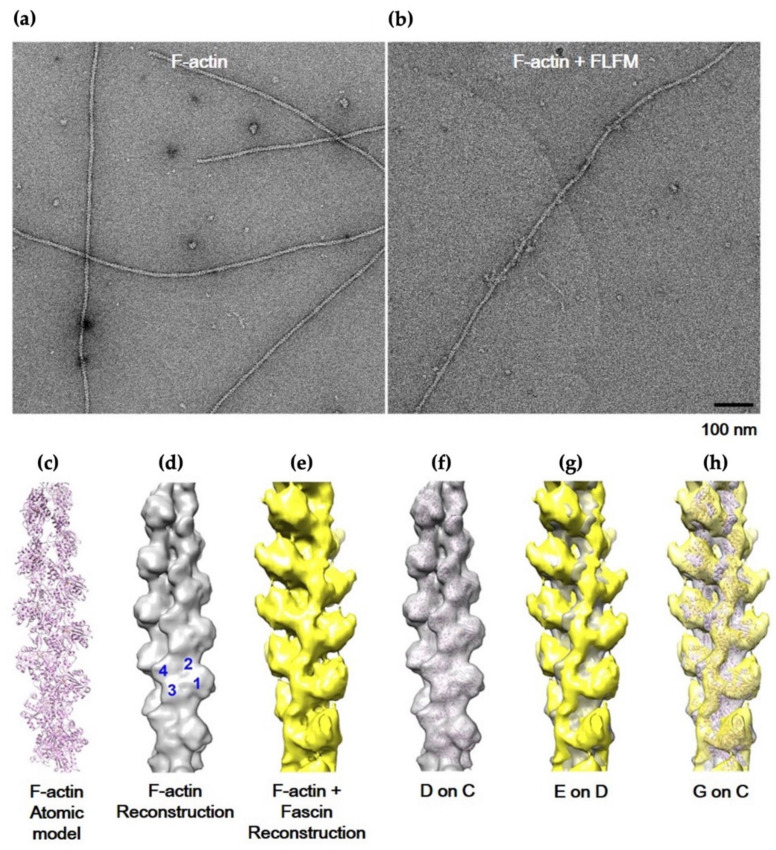
Actin-bundling activity of ∆hydrophobic interaction mutants. (**a**,**b**). Negatively stained EM micrographs showing pure F-actin (phalloidin-treated) and FLF-monomer/F-actin complex (F-actin+FLFM). The scale bar represents 100 nm. (**c**) F-actin atomic structure. (**d**) Surface view of F-actin 3D electron density map, which is reconstructed using actin atomic structure. (**e**) Reconstructed 3D electron density map of fascin-monomer/F-actin complex. (**f**) Superimposition of F-actin atomic model and reconstructed F-actin 3D density map. (**g**) Superimposition of 3D density maps of (**d**,**e**). (**h**) Superimposition of 3D models of (**c**,**g**).

To gain insights into the F-actin-binding model of fascin, IHRSR, which is a specific single-particle analysis of EM for helically ordered protein, was employed with F-actin and FLFM (Figure 6a–h). As a control for the complex of fascin with F-actin, the atomic structure of pure F-actin was processed under similar conditions to those used for the complex (Figure 6d). For the 3D reconstruction of the F-actin–fascin binding model, negatively stained EM micrographs of actin filaments extensively decorated with FLFM were used to extract 8700 short segments (each containing 20 actin molecules). The IHRSR approach allowed us to select 3800 uniformly decorated segments, and these were used to generate a 3D reconstruction of fascin monomer-decorated F-actin (Figure 6e). When the 3D reconstructed electron density map was superimposed to the control pure F-actin 3D model, there was a small amount of additional density on the border of actin subdomain 1 and 2, suggesting that fascin protein may bind to the actin filament through the interaction between the ABS2 of fascin and the pocket area between subdomain 1 and 2 of actin. 

To expand the analysis on the inter-filament distance and bundling mode of fascin protein, electron tomography was also performed by acquiring tomographic tilt series of negatively stained FLF-dimer/F-actin complex (Supplementary Figure S1a–f). For the process, well-ordered, two-stranded bundling filaments showing additional decorated density were selected for 3D reconstruction (Supplementary Figure S1a).

The reconstructed volume of electron tomography has an artifact from mechanical restriction called “missing wedge”. To minimize and overcome this issue, the 3D model of IHRSR was superimposed to the final 3D reconstructed tomogram. This analysis shows that the extra density displayed on the 3D helical reconstruction model was well-fit into the 3D tomography data, and the inter-filament distance within actin bundle was determined to be approximately 5 nm. As shown in Supplementary Figure S1f, the analysis revealed that the fascin dimer uses either side of ABS2 for actin binding, and the dimer is inserted into the actin bundles in a slightly oblique fashion. Based on these EM analyses, we concluded that fascin binds to the border of actin subdomain 1 and 2 via ABS2, and consequently, it leads to the formation of actin bundles through dimerization.

## 4. Discussion

Fascin, which is a 55 kDa actin cross-linking protein, is of great interest because it is expected to play a role in actin cytoskeletal reorganization. Its actin-bundling activity can determine cell migratory and invasive activities in various biomedical areas, including cancer metastasis and neuronal cell development. This protein is well-known as a monomeric protein displaying internal pseudo-symmetry, which allows the protein to bind actin through two structurally related but different actin-binding sites [23]. It is known that fascin has two major actin-binding sites located within β-trefoil domains 1 and 3, and these actin-binding sites have been thought to be conformationally inter-connected to induce actin-bundling activity with monomer form [23]. 

Present results, however, suggested that fascin’s ASB2 actin-binding site, which resides at β-trefoil domains 1 and 2, and the C-terminal dimerization region are important for fascin-dependent actin bundling, presumably through the binding to adjacent actin filaments with each ABS2 of the monomer subunit. As shown in Figure 5b, the actin-bundling activity was lost when the C-terminal 12 residues were deleted (CTD). This truncated mutant has all β-trefoil domains, but the 12 C-terminal residues, which are important for dimerization of the protein, are eliminated. 

While the monomer form of fascin did not show any actin-bundling activity, dimerized fascin protein induced the highly ordered actin-bundling formation (Figure 4). These data imply that the ABS2 in one fascin monomer is not able to induce actin bundling, and the bundling activity requires the dimerization of the protein. This notion was supported by the EM structural analyses with the 3D reconstruction of fascin–F-actin binding and fascin-induced bundling model of fascin. 

The structural analyses revealed that the inter-filament distance within actin bundles formed by fascin dimer was approximately 5 nm (Supplementary Figure S1). This is shorter than reported in a previous report, which shows the average apparent distance between filaments of approximately 8 nm [23]. Although the inter-filament distance estimated in the present study is different, the fascin dimer clearly inserted into the space between the two filaments in a slightly oblique fashion (Supplementary Figure S1).

The present findings suggest that the actin-bundling activity of fascin is accomplished by F-actin binding to ABS2 through dimerization. One of critical questions is whether the dimerization is achieved before actin binding or whether the binding facilitates the dimer formation. Previous studies have shown that actin-binding activity of fascin is regulated by phosphorylation at a serine residue (serine 39), and cell adhesion to extracellular matrix macromolecules differentially regulates the phosphorylation state of fascin [6,26]. In this regard, further in vivo study is required to clarify relationship between phosphorylation and dimerization of fascin and the molecular mechanism involved in the dimerization-dependent actin-binding and -bundling activity of fascin through reconstitution of various mutant cells. 

Due to the critical roles of fascin in cell migration, its importance is emphasized in association with various diseases [27,28,29]. The well-studied pathological role of fascin is in cancer malignancy, and it is a common biomarker for aggressive carcinomas [27]. Recently, a fascin inhibitor is in Phase I clinical trials as an anti-tumor agent for metastatic colorectal cancer [30]. In addition to that, fascin is also involved in other pathological conditions, including would healing and retinal and neurological disorders [31,32,33,34]. Although several studies have been conducted to investigate the functions of fascin in various pathological conditions, how this protein and its isoforms contribute to diseases is not fully determined yet. Thus, it is anticipated that understanding the structural basis of fascin-dependent actin bundling will provide an important clue for the design of new fascin inhibitors that can be used for various related diseases.

## Supplementary Materials

The following supporting information can be downloaded at: https://www.mdpi.com/article/10.3390/life12060843/s1, Figure S1: Actin bundling activity of ∆hydrophobic interaction mutants.
