# Supplementary material for: Structural Analysis of Human Fascin-1: Essential Protein for Actin Filaments Bundling
Source: Life (Basel). 2022 Jun 6;12(6):843. doi: 10.3390/life12060843 (PMC9224989; doi:10.3390/life12060843)
Supplement: Supplementary file 1 [file life-12-00843-s001.zip › SupplementaryFigure1.pdf]

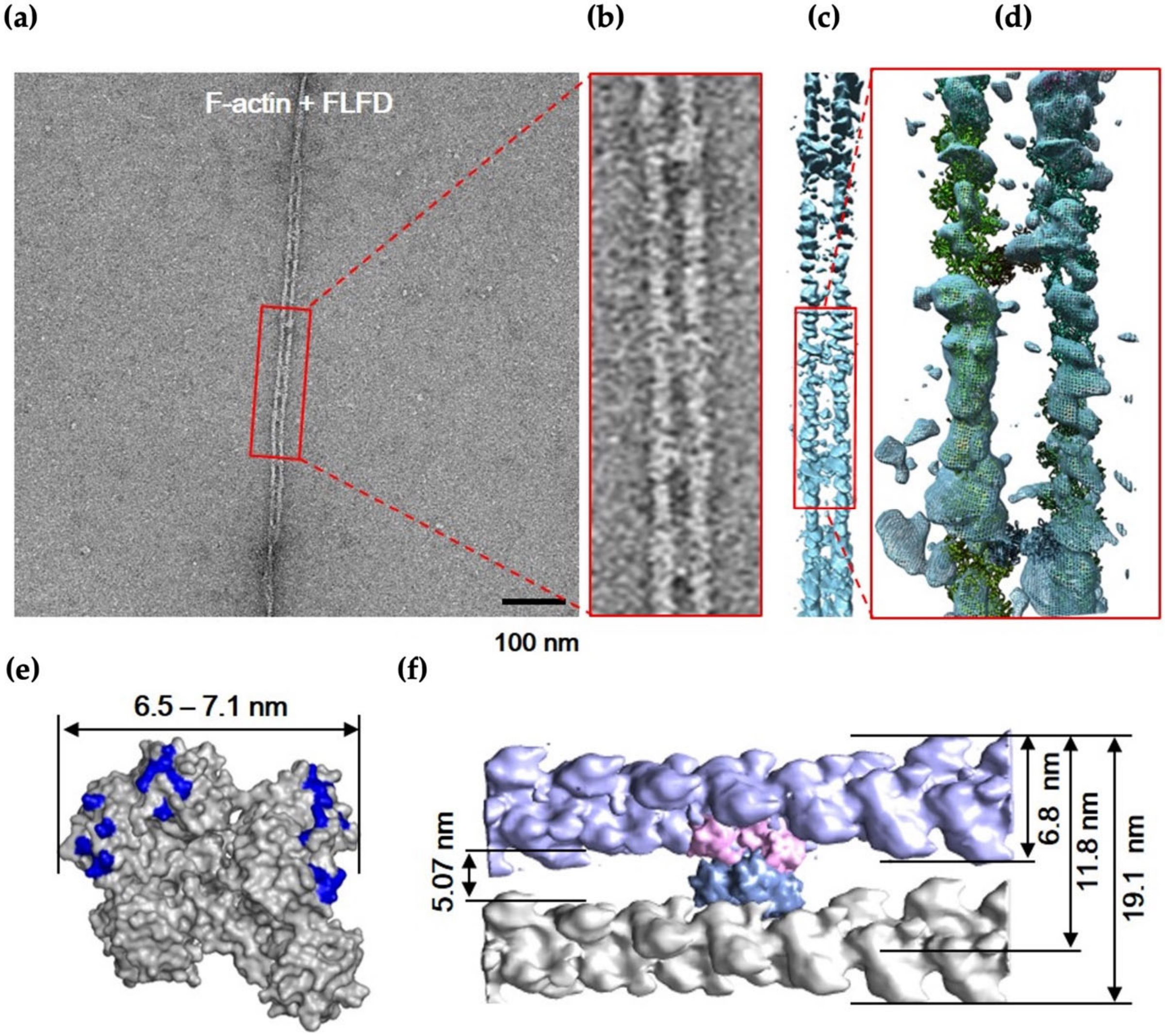

Supplementary Figure S1. Actin bundling activity of ∆hydrophobic interaction mutants. **(a)** Negatively stained EM field of double stranded F-actin filament induced by FLF-dimer (FLFD). **(b)** Enlarged view of FLF-dimer decorated region. **(c)** Reconstructed 3D electron tomogram of double-stranded FLF-dimer/F-actin complex. **(d)** Superimposition of atomic structure of F-actin, 3D helical reconstruction model and FLF-dimer to the reconstructed 3D tomogram of FLF-dimer/F-actin complex. **(e)** Dimer model of fascin. The inter-molecular distance is indicated by two-head arrow. **(f)** Surface view of atomic model fitted to electron density map.
